# A Novel Sub-Lineage of Chikungunya Virus East/Central/South African Genotype Indian Ocean Lineage Caused Sequential Outbreaks in Bangladesh and Thailand

**DOI:** 10.3390/v12111319

**Published:** 2020-11-17

**Authors:** Juthamas Phadungsombat, Hisham Imad, Mizanur Rahman, Emi E. Nakayama, Sajikapon Kludkleeb, Thitiya Ponam, Rummana Rahim, Abu Hasan, Kanaporn Poltep, Atsushi Yamanaka, Wasin Matsee, Watcharapong Piyaphanee, Weerapong Phumratanaprapin, Tatsuo Shioda

**Affiliations:** 1 Mahidol-Osaka Center for Infectious Diseases, Faculty of Tropical Medicine, Mahidol University, Bangkok 10400, Thailand; juthamasps@gmail.com (J.P.); hisham.a.imad@gmail.com (H.I.); kanaporn.pol@gmail.com (K.P.); knmya@biken.osaka-u.ac.jp (A.Y.); 2 Hospital for Tropical Diseases, Faculty of Tropical Medicine, Mahidol University, Bangkok 10400, Thailand; sajikaponk@gmail.com (S.K.); thitiya.pon@mahidol.ac.th (T.P.); wasin@thaitravelclinic.com (W.M.); watcharapong.piy@mahidol.ac.th (W.P.); weerapong.phu@mahidol.ac.th (W.P.); 3 Apollo Hospitals Dhaka (presently Evercare Hospital Dhaka), Dhaka 1229, Bangladesh; mizanur.rahman@evercarebd.com (M.R.); rummana.rahim@evercarebd.com (R.R.); rasel.hasan@evercarebd.com (A.H.); 4 Research Institute for Microbial Diseases, Osaka University, Osaka 565-0871, Japan; emien@biken.osaka-u.ac.jp; 5 Department of Clinical Tropical Medicine, Faculty of Tropical Medicine, Mahidol University, Bangkok 10400, Thailand

**Keywords:** chikungunya virus, East/Central/South African genotype, Indian Ocean lineage, outbreaks, molecular clock analysis, Bangladesh, Thailand, mosquito

## Abstract

In recent decades, chikungunya virus (CHIKV) has become geographically widespread. In 2004, the CHIKV East/Central/South African (ECSA) genotype moved from Africa to Indian ocean islands and India followed by a large epidemic in Southeast Asia. In 2013, the CHIKV Asian genotype drove an outbreak in the Americas. Since 2016, CHIKV has re-emerged in the Indian subcontinent and Southeast Asia. In the present study, CHIKVs were obtained from Bangladesh in 2017 and Thailand in 2019, and their nearly full genomes were sequenced. Phylogenetic analysis revealed that the recent CHIKVs were of Indian Ocean Lineage (IOL) of genotype ECSA, similar to the previous outbreak. However, these CHIKVs were all clustered into a new distinct sub-lineage apart from the past IOL CHIKVs, and they lacked an alanine-to-valine substitution at position 226 of the E1 envelope glycoprotein, which enhances CHIKV replication in *Aedes albopictus*. Instead, all the re-emerged CHIKVs possessed mutations of lysine-to-glutamic acid at position 211 of E1 and valine-to-alanine at position 264 of E2. Molecular clock analysis suggested that the new sub-lineage CHIKV was introduced to Bangladesh around late 2015 and Thailand in early 2017. These results suggest that re-emerged CHIKVs have acquired different adaptations than the previous CHIKVs.

## 1. Introduction

Chikungunya virus (CHIKV) is a mosquito-borne virus transmitted via *Aedes* mosquitoes that causes a febrile illness accompanied by rash and arthralgia. CHIKV belongs to genus *Alphavirus*, family *Togaviridae,* and it contains a single-stranded RNA genome of approximately 11–12 kb with two open reading frames (ORFs) flanked by untranslated regions (UTRs) at the 5′ and 3′ ends. The CHIKV genome encodes two polyproteins that are subsequently processed into four nonstructural and four structural proteins. Although the first CHIKV was isolated in 1952 in Tanzania, both clinical records in the 18th and 19th centuries and molecular clock analyses of current CHIKV genomes suggest that this virus has existed for 300 years [1,2]. At present, CHIKV is phylogenetically classified into three genotypes reflecting the geographical locations where the respective strains were first isolated, East/Central/South African (ECSA), West African (WA), and Asian. CHIKV is transmitted by *Aedes* mosquitoes that are found in tropical and temperate regions. In particular, *Ae. aegypti* is predominant in tropical areas in East Africa, South Asia, and Southeast Asia where a re-emergence of CHIKV has occurred [3].

The emergence of new lineages has been common in recent decades. In 2004, the ECSA-genotype CHIKV circulated in *Ae. aegypti* in coastal Kenya and spread to the Comoros and other islands in the Indian Ocean, India, and Sri Lanka, and it has since diverged into a new lineage called the Indian Ocean Lineage (IOL) [2]. Shortly thereafter, IOL CHIKV reached Southeast Asia, particularly Singapore, Malaysia, and Thailand, and it caused large epidemics that resulted in over a million suspected CHIKV cases [4]. An alanine-to-valine mutation at position 226 within the E1 glycoprotein confers enhanced virus replication in *Ae. albopictus* and has been implicated in the widespread circulation of CHIKV in Asia and even in temperate climate countries such as Italy and France [4,5,6]. In December 2013, Asian-genotype CHIKV that originated in Micronesia in the Pacific was introduced into the Caribbean Sea, which is a region with naïve immunity to CHIKV and abundant *A. aegypti*. The outbreak started in St. Martin, was transmitted to other islands, and finally rapidly spread throughout Central and South America during late 2014–2016. These CHIKVs have been characterized as the Caribbean outbreak lineage (COL) [7]. Moreover, ECSA-genotype CHIKV also appeared in Brazil in 2014 [8].

**Figure 1:**
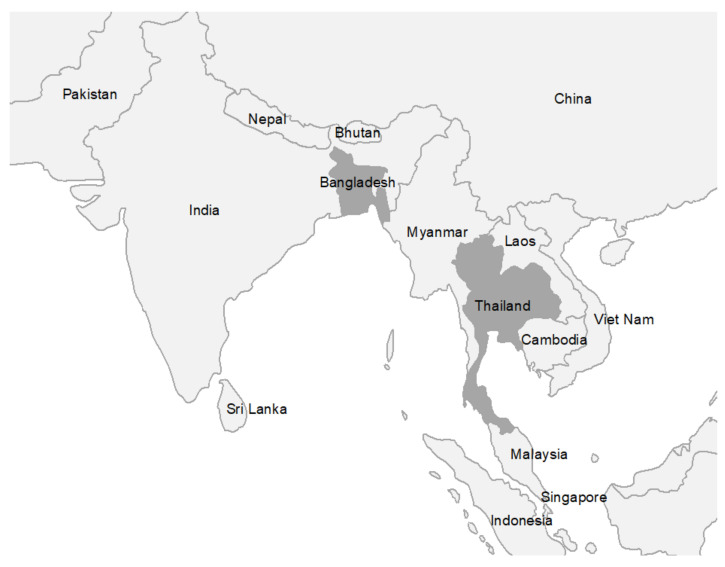
Locations of chikungunya virus (CHIKVs) collection in the present study. Bangladesh and Thailand, where CHIKV sera were collected, are indicated in the dark grey shade area.

In 2016, another CHIKV outbreak started in northern India [9,10] and Pakistan, and it spread to Bangladesh in 2017 [11] and even Kenya and Italy in 2016–2017 [12,13,14]. A re-emergence of CHIKV has also been reported in Thailand since 2018 until present [15]. In the present study, to investigate the evolutionary relationships and genetic diversity of CHIKVs that have re-emerged since 2016, we conducted a phylogenetic analysis using the complete coding regions of CHIKVs from South and Southeast Asia, namely Bangladesh and Thailand (Figure 1), collected in 2017 and 2019, respectively, together with public sequences from GenBank. The results revealed that both CHIKV sequences from Bangladesh and Thailand belonged to ECSA-IOL CHIKV. However, these sequences clustered in a distinct clade apart from the previous IOL CHIKV clade from 2004–2013, forming a new sub-lineage. Our molecular clock analysis suggested that the new sub-lineage IOL emerged in 2008 and was introduced into Bangladesh around 2015, and then into Thailand in early 2017.

## 2. Materials and Methods

### 2.1. CHIKV Samples

This study was conducted in accordance with the Declaration of Helsinki and was exempt from obtaining participants’ consent since only leftover specimens were used after anonymization. All viruses analyzed in the present study are listed in Table 1. Twenty acute febrile cases that were serum-positive for CHIKV by real-time RT-PCR were collected at the Apollo Hospitals Dhaka in Dhaka city, Bangladesh, in 2017 [11,16]. This study proposal was approved by the Research and Ethics Committee of Apollo Hospitals Dhaka (ERC 16/2018–3). Fourteen CHIKV-positive sera samples were also obtained from the Hospital for Tropical Diseases in Bangkok, Thailand, in 2019. This study proposal was approved by the Research and Ethics Committee of the Faculty of Tropical Medicine, Mahidol University (FTM ECF-035-01). High viral load (C_t_ value less than 20) sera were directly used for RT-PCR amplification of the CHIKV genome, while the other sera were subjected to virus isolation in the C6/36 mosquito cell line cultivated in L-15 medium (Hyclone, UT, USA) supplemented with 2% fetal bovine serum (Hyclone, UT, USA). Inoculated cells were incubated at 28 °C until the cytopathic effect appeared, and culture supernatants were subjected to real-time RT-PCR to confirm successful virus isolation. One laboratory CHIKV strain (CP10) was isolated in 2010 [17].

### 2.2. CHIKV Genome Sequencing

Either 70 μL of sera or cultured supernatant were used for RNA extraction by QIAamp viral RNA mini kits (Qiagen, Hilden, Germany). Two overlapping amplified DNA fragments covering the full genome of CHIKV were prepared, quantified, and normalized to 0.2 ng/μL using a Qubit fluorometer (Themo Fisher Scientific, MA, USA). Five μL of the amplified fragments were processed for library preparation by an Illumina Nextera XT kit (Illumina, San Diego, CA, USA) and the paired-end of the 2 × 250 bp sequencing reaction was conducted using the Miseq platform (Illumina, San Diego, CA, USA) according to a previously published protocol [18]. The forward and reverse short reads were imported to CLC Genomics Workbench software version 20 (Qiagen, Aarhus, Denmark) and then aligned to the MF773566-Bangladesh 2017 sequence [19] using map reads to the reference command. Finally, the consensus sequence was extracted.

### 2.3. Nucleotide Sequence Accession Numbers

All sequences generated in the present study were deposited to GenBank under accession numbers LC580236–LC580255 and LC580256–LC580270 for Bangladesh and Thailand sequences, respectively.

### 2.4. Phylogenetic Analyses

To classify the CHIKV genotype of the obtained sequences in the present study, the 35 newly generated sequences were combined with reference CHIKV sequences downloaded from the NIAID Virus Pathogen Database and Analysis Resource (ViPR; http://www.viprbrc.org/) [20] and GenBank (https://www.ncbi.nlm.nih.gov/genbank/) representing 3 genotypes: WA, Asian, and ECSA viruses [2,5,21,22] (Table S1). All sequences were aligned with Muscle implemented in AliView v1.26 [23]. The aligned sequences were trimmed to ORF regions, and ambiguous regions in UTRs were removed. The CHIKVs genotype maximum-likelihood tree (ML tree) was constructed in IQ-TREE under GTR+F+I and 1000 replicates of ultrafast bootstrap [24,25,26].

To further investigate the viral evolution with a Bayesian phylogenetic molecular clock approach, a second dataset of ECSA-CHIKV was prepared covering the geography and time spanning from the 1950s to the latest outbreak in the present study. This ECSA-CHIKV dataset was tested for the presence of recombination using GARD implemented in the Datamonkey server (https://www.datamonkey.org/) before proceeding to the construction of the initial ML tree, under GTR+F+I+G4 and 1000 replicates of bootstrap, to determine the evolutionary temporal signal using Tempest v1.5.3 [27]. To reconstruct the time-scale phylogeny, Bayesian Markov Chain Monte Carlo (MCMC) sampling was undertaken in the BEAST v1.10.4 package [28]. The best-fit model of a relaxed uncorrelated log normal clock and the Bayesian coalescent tree prior of Bayesian Skygrid under SRD06 nucleotide substitution was chosen by comparison with the other models by path sampling (PS) and stepping-stone sampling (SS) methods with 50 path steps of 1 million iterations and log every 1000 (Table S2) [29,30,31]. The grid point was set at 75 as predicted by the intercept of root-to-tip regression of the initial ECSA-CHIKV ML tree. Each sequence was tagged to a geographic region according to the collected location. The four independent runs of MCMC were done for 150 million generations each, with sampling every 15,000 generations. The running speed was enhanced using BEAGLE. All runs were combined to achieve good mixing of convergence in LogCombiner v1.10.4. The trace was accessed in Tracer v1.7.1, and then a 10% burn-in was removed. The maximum clade credibility (MCC) tree was generated using TreeAnnotator v1.10.4 and visualized in FigTree v1.4.4.

To determine lineage specific amino acid mutations, the corresponding amino acids of the ECSA-CHIKV nucleotide alignment were aligned in AliView v.1.26. Amino acid positions were annotated following HM045811-Tanzania-1953 CHIKV.

### 2.5. Selection Analyses

The two individual ORFs of nonstructural polyprotein and structural polyprotein corresponding to the ECSA CHIKV dataset, and the newly generated CHIKV dataset was applied for selection pressure analysis implemented in the DataMonkey server (http://datamonkey.org/) by using three methods: mixed-effects model of evolution (MEME), fast, unconstrained Bayesian approximation (FUBAR), and fixed-effect likelihood (FEL) for the site-specific selection [32,33,34,35].

## 3. Results

### 3.1. ECSA-IOL CHIKVs in Bangladesh and Thailand During 2017–2019

**Figure 2:**
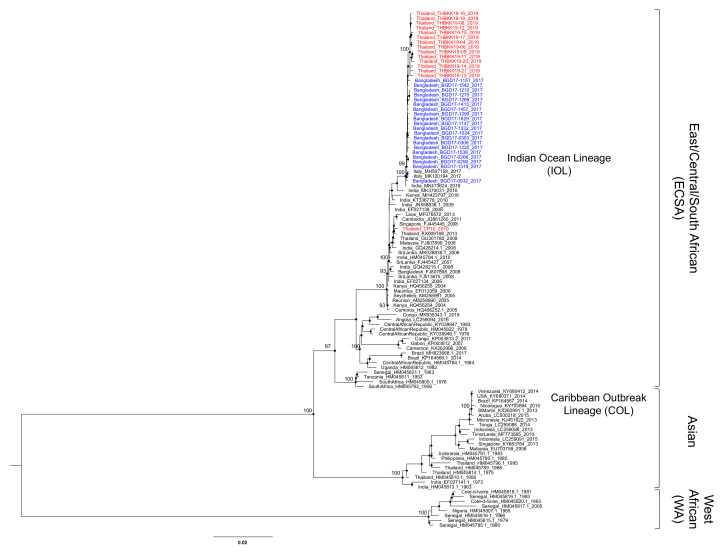
Genotype classification of CHIKV. The maximum-likelihood tree of open reading frames (ORFs) were constructed using GTR+F+I with 1000 ultrafast bootstrap replications. The Bangladesh and Thailand sequences obtained in the present study are labeled with blue and red, respectively. The CHIKV genotypes are indicated to the right. The recent lineages are shaded in the gray box and indicated to the right. Bootstrap support values exceeding 80% are shown as black nodes and the actual values are indicated only in key important nodes.

Twenty CHIKV coding sequences in Dhaka in 2017, 14 CHIKV sequences in Bangkok in 2019, and 1 CHIKV sequence in 2010 were aligned with the reference sequences of three genotypes: WA, ECSA, and Asian. The ML phylogenetic tree of the resultant alignment showed three distinct monophyletic clades of each genotype with strong bootstrap support of 100 (WA), 97 (ECSA), and 100 (Asian) (Figure 2). A CHIKV ECSA-IOL outbreak started in 2005 in the Indian Ocean islands and then spread to South and Southeast Asia, while an Asian-COL outbreak started in 2013 in the Pacific islands and then spread to the Caribbean islands and Americas. Our 35 new CHIKVs belonged to genotype ECSA-IOL, which contains several clusters from the Indian Ocean islands, the Indian subcontinent, and Southeast Asia. However, except for the CP10 strain that was collected during the previous outbreak in 2010, all of our Bangladesh and Thailand sequences formed a distinct monophyletic cluster apart from the previous IOL clusters with bootstrap support of 100, and the most closely related virus was the Indian strain reported in 2016 (MK473624). The recent viruses reported from Italy in 2017 were also found in this cluster.

### 3.2. Evolution of the ECSA-CHIKV IOL Sub-Lineage

**Figure 3:**
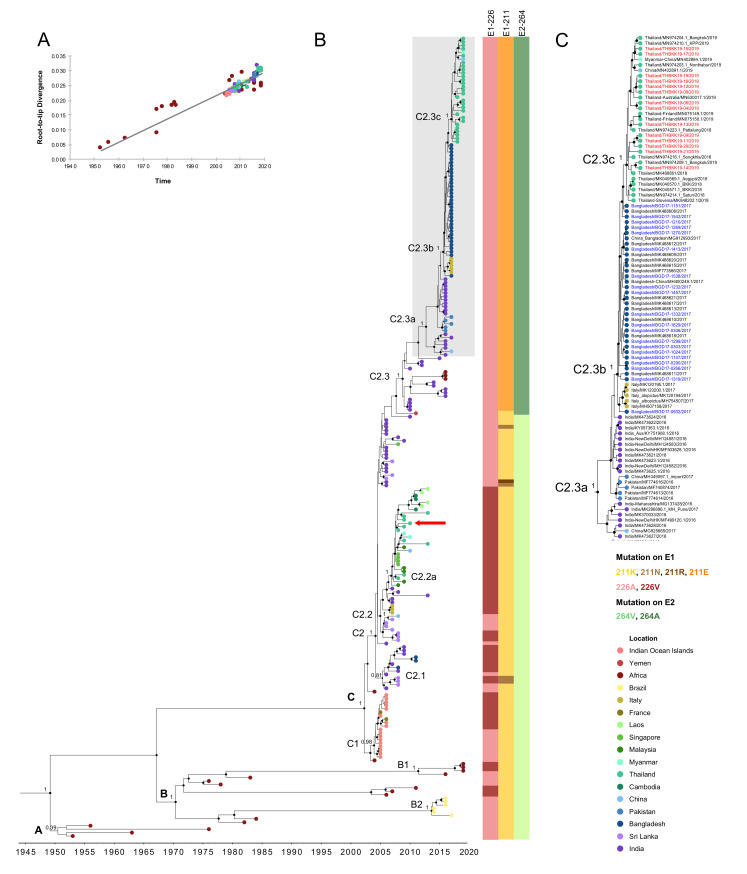
Molecular clock analysis of ECSA-genotype CHIKV. (**A**) Temporal signal analysis of regression of root-to-tip divergence against date. The color marker represents the location indicated in the left panel. (**B**) Maximum clade credibility (MCC) tree of East/Central/South African (ECSA)-genotype CHIKV ORFs. The timescale in years is shown in the *x*-axis at the bottom. Black nodes are shown only for those with the posterior probability (PP) more than 0.8. Yellow triangles indicate the key nodes, and PP values are shown adjacent to the corresponding key nodes. CP10 is shown by a red arrow. The round tip color of each sequence corresponds to the location indicated in panel C. (**C**) Magnification of a gray-shaded area in B including recent India, Bangladesh, and Thailand clades. The Bangladesh and Thailand sequences obtained in the present study are labeled with blue and red, respectively. Sequences are designated in the format of country/accession number/year of collection. The amino acid mutations specific to the Indian Ocean Lineage (IOL) correspond to their sequences shown next to the sequence tip of the MCC tree in panel B and indicated in the right panel.

To investigate the genetic relationships among the previous and recent ECSA-IOL CHIKVs, we collected additional sequences of ECSA-IOL from recent reports and subjected them to molecular clock analysis. To estimate the time of the most recent common ancestor (tMRCA) and the evolution rate of the new ECSA-IOL CHIKV sub-lineage circulating in 2016–2019, the expanded dataset of the CHIKV ECSA genotype (*n* = 233) was analyzed using Bayesian phylogenetic inference. The alignment of the sequence dataset showed no recombination among the collected CHIKV sequences. Then, we constructed an initial ML tree for root-to-tip analysis. The linear regression divergence against collection time exhibited a positive temporal signal of 0.88. By this method, the tMRCA was dated at 1945.57, and the substitution rate was estimated at 3.95 × 10^−4^ substitutions/site/year (s/s/y) (Figure 3A). The relaxed uncorrelated lognormal clock and Skygrid, the best-fit model, was used for estimation (Table S2). The timescale MCC tree showed that the root tMRCA of ECSA was estimated to be around 1949.17 (95% highest posterior density (HPD): 1944.14–1957.42) (Figure 3B and Figure S1). The lineages, sub-lineages, and clades were arbitrary named and are described in Table 2 according to the defined nodes in Figure 3B.

As shown in the CHIKVs derived from node A in Figure 3B, ECSA CHIKVs that circulated around the 1950s–1970s in Africa first emerged in 1950.48 (95%HPD: 1948.58–1952.53). Around 1970.39 (95%HPD: 1967.68–1973.27), CHIKVs derived from node B diverged into two lineages B1 and B2. The tMRCA of recent node B1 viruses in Angola and Congo was estimated to be 2011.39, while that of node B2 viruses in Brazil was 2013.66. The major expansion of ECSA from Africa to new territories in Asia, which was established as IOL (referring to node C), was dated to approximately 2002.26 (95%HPD: 2001.14–2003.59). This IOL was split into two sub-lineages: C1) CHIKVs from Indian Ocean islands and C2) those from India, Sri Lanka, Bangladesh, and Southeast Asia showing similar tMRCA at 2003.92 (95%HPD: 2003.43–2004.45, PP = 0.98) and 2004.16 (95%HPD: 2003.52–2004.88, PP = 1). The phylogenetic tree suggested that the ancestral strains of these sub-lineages were closely related to CHIKVs detected in coastal Kenya in 2004 (HQ456254 and HQ456255) [36]. The sub-lineage of the Indian subcontinent and Southeast Asia had a large branch consisting of several descendant clades including the Southeast Asia clade C2.2a that emerged in 2006.82 (95%HPD: 2006.42–2007.25). In addition, our strain CP10 collected in 2010 from Ratchaburi, Thailand also fell into this clade clustered with other Thailand CHIKVs with a similar collection date.

In contrast, not all but some Indian CHIKV sequences clustered into a separate clade (PP = 1) derived from node C2.3. This clade consisted of the CHIKV sequences from India in 2010-2012 accompanied by recent sequences after 2016 from India, Pakistan, Bangladesh, Italy, and Thailand. The tMRCA of these CHIKVs was dated at 2008.75 (95%HPD: 2008.18–2009.37). Bangladesh and Thailand sequences obtained in the present study belonged to this clade. Our Bangladeshi CHIKV sequences in 2017 clustered with other Bangladeshi and Italian CHIKV strains in the same collection year. Their tMRCA was estimated to be 2015.61 (95%HPD: 2015.25–2016.01, PP = 1) at node C2.3b, and they shared a common ancestral strain with the Indian strain in 2016 (MK473624) [9]. Furthermore, consequent CHIKV sequences descended from node C2.3c, including our Thailand and other Thailand strains in 2018-2019 together with CHIKVs from Myanmar and China, formed a monophyletic clade (PP = 1), and shared a tMRCA at 2016.98 (95%HPD: 2016.73–2017.25, PP = 1).

### 3.3. Signature Mutations Related to CHIKV ECSA-IOL Sub-Lineages among CHIKV Outbreak Strains

The tree topology of ECSA-IOL CHIKV showed that several distinct lineages diverged during 2005–2019. Therefore, we further examined the nonsynonymous mutations during the evolution of the lineages, especially those of the previous IOL outbreak in 2005 and the recent IOL outbreak. An alanine-to-valine substitution at residue 226 of the envelope E1 protein (E1-A226V) has been reported to facilitate CHIKV replication in *Ae. albopictus*. Herein, this mutation was observed in certain sequences of node C1-viruses (IOL-Indian Ocean islands), node C2.1-viruses (IOL-Indian subcontinent), and C2.2-viruses (IOL-Indian subcontinent/SEA). Remarkably, all the C2.2a viruses obtained during the previous Southeast Asian outbreak including in Thailand carried E1-226V (Figure 3B and Table 3). However, E1-A226V was not detected, but E1-226A was found in all sequences in the current outbreak node C2.3 viruses (northern Indian subcontinent/SEA). Furthermore, the descendant sequences in node C2.3 viruses exhibited unique E1-K211E and E2-V264A substitutions, which are novel lineage-specific substitutions, in all the current outbreak strains in India and Pakistan in 2016, in Bangladesh in 2017, in Italy in 2017, and in Thailand in 2019 (Figure 3 and Table 3). In addition, the viruses derived from node C2.3a, i.e., India 2016, Pakistan 2016, Bangladesh 2017, and Thailand 2019 (Figure 3C and Table 3), shared five substitutions: nsP2-H130Y, nsP2-E145D, nsP4-S55N, nsP4- R85G, and E1-I317V. Moreover, the descendant clade defined by node C2.3b shared two more amino acid substitutions, nsP3-D372E and E2-G205S. Interestingly, nsP2-793V/A was found in Bangladeshi strains, while nsP2-V793A and nsP2-N495S were found in all sequences of the Thailand clade defined by node C2.3c, including Thailand, Myanmar, and China strains.

### 3.4. Selection Analyses

To identify amino acids under selection pressure within the ECSA-CHIKV, analyses using Datamonkey methods FEL, FUBAR, and MEME were conducted by aligning individual coding regions of the nonstructural polyproteins and structural polyproteins of all sequences used in Figure 3 (Table 4). A subset of the sequences obtained in the present study was also used to detect recent evolution of ECSA-CHIKV (Table 4). Residues nsP1-171, nsP3-217, and E1-211 in the total ECSA dataset and nsP3-58 and C-73 in the recent ECSA dataset were shown to be under positive selection pressure at statistically significant *p*-values of <0.05 in FEL and MEME and PP > 0.9 in FUBAR.

## 4. Discussion

The emergence of the ECSA CHIKV lineage IOL caused one million cases over a decade. Sequential outbreaks are still being reported in the Indian subcontinent. Bangladesh, located to the northeast of India, faced a second large outbreak in 2017, when the highest numbers of confirmed cases were reported during May–July [37]. In the present study, we examined CHIKV-positive serum from Dhaka during July–December 2017 [11]. Later in 2018–2019, CHIKV transmission was observed in nearby regions simultaneously, and the number of CHIKV cases noticeably increased in travelers and locals in Myanmar and especially Thailand [19,38,39,40]. According to the national surveillance of Thailand, the numbers of confirmed cases were 10, 3,580, and 11,721 in 2017, 2018, and 2019, respectively [15]. We also examined CHIKV-positive serum collected at the Hospital for Tropical diseases, Bangkok, in October 2019.

Our results revealed a new distinct IOL sub-lineage separate from the previous IOL that previously caused outbreaks in the Indian Ocean, Indian subcontinent, and Southeast Asia in the last decade. According to our estimated tMRCA, this new IOL sub-lineage originated from India CHIKV, which circulated during 2008–2016. Then, the virus was introduced to Pakistan in early 2015, to Bangladesh in mid-2015, and reached Thailand in 2017. Furthermore, this virus was also detected in Italy around 2016. In contrast, the previous emergence of IOL originated from coastal Kenya, was split into two different sub-lineages in the Indian Ocean islands and Indian subcontinent in 2003, and the Indian subcontinent sub-lineage subsequently reached Southeast Asia in late 2006. The evolution rate of our new sub-lineage of IOL in the present study, 6.29 × 10^−4^ (1.57 × 10^−4^–1.30 × 10^−3^ s/s/y), was similar to that of the previous IOL outbreak in 2004–2013, 7.51 × 10^−4^ (6.67 × 10^−4^–8.46 × 10^−4^ s/s/y) [41]. The evolution rate in the Bangladesh clade was 6.79 × 10^−4^ (1.75 × 10^−4^–13.76 × 10^−4^ s/s/y), and that in the Thailand clade was 1.08 × 10^−3^ (3.07 × 10^−4^–2.04 × 10^−3^ s/s/y), although the broad HPD range might be due to a low number of available sequences in the present study.

Interestingly, among this new IOL sub-lineage, the adaptive mutation E1-A226V, which facilitates virus replication in *Ae. albopictus* [5,42], was not detected. Instead, another mutation, E1-K211E, was detected in all sequences in this sub-lineage. *Ae. aegypti* and *Ae. albopictus* coexist in India, where a mixture of IOL variants with E1-226A or 226V and E1-211K or 211E has been observed. The epidemics in India were initiated in 2005-2006 and were caused by either IOL E1-226A or 226V variants. However, later studies in 2007-2010 reported that IOL E1-226A, a wild-type or reverted strain, was the major strain in India [21,22,43,44]. The IOL E1-226V variant had been dominant in states such as Kerala where *Ae. albopictus* is abundant [22,45,46]. Later, apparently both E1-226V and E1-226A viruses had continued to spread in Kolkata of West Bengal. The E1-226V variant was spotted in rural areas, while the E1-226A virus was found in urban areas, where *Ae. albopictus* and *Ae. aegypti* are abundant, respectively [47]. Remarkably, in 2008, the IOL E1-226V variant successfully spread to Singapore and subsequently to other Southeast Asian countries where *Ae. albopictus* served as the primary vector [48,49]. In addition, an expansion of IOL E1-226A virus was detected in northern India and was supposedly related to the outbreaks in New Delhi in 2010 and 2016 [9,50].

In addition to E1-K211E, E2-V264A was first detected in 2010 in India in Kerala and New Delhi, and also from *Ae. aegypti* in Yemen [9,45,51]. They are also specific to the new IOL sub-lineage that emerged around mid-2008. An early appearance of these two substitutions was detected in Southern India around late 2009-2010 [44], followed by New Delhi in 2010 and 2016 [9,10]. Additionally, in 2010-2011, in Uttar Pradesh, a state located in northern India, a sequence determination of the E1 region in mosquitoes showed the co-existence of E1-211K and E1-211E variants in the background of E1-226A in *Ae. aegypti* and E1-211K in the background of E1-226V in *Ae. albopictus* [52]. These substitutions of E1-K211E and E2-V264A were commonly observed in the new IOL sub-lineage viruses. The present study also showed that the codon 211 of E1 was under positive selection. Accordingly, genetically engineered mutations of E1-K211E and E2-V264A in the background of E1-226A confer increased infection, dissemination, and transmission in *Ae. aegypti* [53].

In mid-2015, the new IOL CHIKV sub-lineage carrying E1-K211E and E2-V264A in the background of E1-226A was introduced to Bangladesh and replaced the old IOL CHIKV that was reported earlier in 2008–2011 in rural areas such as the Dohar sub-district in Dhaka and the Chapainababganj district located in northwestern Bangladesh [54,55]. This new sub-lineage caused the largest CHIKV outbreak in 2017. More than one thousand confirmed cases were reported in Dhaka city, and numbers of cases peaked in July during the monsoon season [56,57]. Although *Ae. albopictus* had been identified as the main vector for the previous outbreak in Dohar, Dhaka district [55], there has been no report on the mosquito species vector for the new IOL CHIKV sub-lineage. However, a previous study examining approximately 12,000 larvae and pupae sampled from households in Dhaka city during the monsoon in 2011–2013 showed that the numbers of *Ae. aegypti* were four times those of *Ae. albopictus* [58], suggesting that *Ae. aegypti* might be the transmission vector for the new sub-lineage of CHIKV IOL. Our Bangladesh CHIKVs were more closely related to those identified in CHIKV meningitis collected in Dhaka rather than those from other regions of Bangladesh [59]. A minor polymorphic mutation, V58I in nsP3, was observed among Bangladeshi CHIKVs only in BGD17-0303, -1024, -1147, -1299, -1332, -1629 and MK468610, MK468613, MK468616, MK468618, MK468622, and MK468625 [59]. In addition, Bangladeshi CHIKVs were also very similar to CHIKV strains reported in travelers and exported to China and Australia in 2017 [19,60], the Italian CHIKVs in 2017, and Thailand CHIKVs in 2018-2019. These closely related viruses shared the new mutations of D372E in nsP3 and G205S in E2, which were not detected in the Indian CHIKV 2016 (MK472624), the most related and earliest strain of recent clades circulated after 2017 of this new sub-lineage.

Concerning Italy, the first CHIKV outbreak in 2007 was caused by the IOL of E1-A226V that was transmitted by *Ae. albopictus* [61]. In 2017, the new CHIKV sub-lineage of IOL was reintroduced to Italy [13]. Our analysis revealed that Italian 2017 CHIKVs were closely related to those in Bangladesh, with E1-K211E and E2-V264A in the background of E1-226A. A recent study on the vector competence of these two IOL sub-lineages of CHIKV in *Ae. albopictus* showed that both had comparable infection and transmission rates [62]. It is tempting to speculate that the acquisition of efficient growth capability in both *Ae. albopictus* and *Ae. aegypti* allowed CHIKV to spread over a wide area.

As mentioned before, the national surveillance conducted by the Bureau of Epidemiology, Department of Disease Control, Ministry of Public Health has reported that CHIKV outbreaks have been ongoing in Thailand [15]. Sporadic suspected CHIKV cases have been routinely monitored through mid-2018. The rise of suspected CHIKV cases was noticed in the most southern Thailand during mid-2018 to the end of 2019, and they hit a peak in December 2018-January 2019 with nearly 3000 suspected cases. Then, CHIKV spread to the upper region in western Thailand during mid-2019 to the end of 2019. Similarly, in Bangkok from July 2019 to present, the numbers of CHIKV suspected cases peaked in October and November 2019 with 396 and 516 suspected cases, respectively. The total number of suspected CHIKV cases reached 11,484 overall in 2019 in Thailand. At present, CHIKVs have been spreading to northern and eastern Thailand. Our Thailand CHIKVs analyzed in the present study were collected in Bangkok during this ongoing outbreak in October 2019. These Bangkok CHIKVs were a new sub-lineage of IOL, which is closely related to and clustered together with southern Thailand strains in 2018, a Myanmar strain in 2019, and Australian, Slovenian, Finland, and Chinese strains imported from Thailand. In addition to new IOL-specific mutations mentioned before, these viruses contained additional polymorphic mutations of nsP3-N495S and Capsid-K73R. An investigation of *Ae. aegypti* collected from all over Thailand revealed that around 16% of field-caught *Ae. aegypti* in Bangkok are CHIKV PCR positive [63]. The rate of CHIKV PCR-positive *Ae. aegypti* was 34% in Prachuap Khiri Khan, a province in the southwestern part of Thailand, which is consistent with the numbers of suspected CHIKV cases from the surveillance as mentioned before. Furthermore, the CHIKV envelope regions in all these mosquitoes are characterized by E1-K211E, E2-V264A, and E1-226A [63]. In addition, new sub-lineage IOL CHIKVs were detected in small numbers of mosquitoes in Song Khla, Krabi, and even in northeastern regions such as Nong Khai province [63]. Unfortunately, all of the Thailand CHIKVs analyzed in the present study were collected in Bangkok, and thus, we could not see the old sub-lineage of IOL E1-A226V, which shows restricted transmission in mosquitoes in northern regions of Thailand such as Chiang Rai, Chiang Mai, and Ubon Ratchathani [63]. Thailand has abundant *Ae. aegypti* and *Ae. albopictus.* In a previous IOL outbreak in Thailand in 2009, the principal vector was *Ae. albopictus* [64]. Although there is no direct evidence of CHIKV transmission by this mosquito species in Thailand at present, it would be interesting to further investigate the vector competence of *Ae. albopictus* for the new sub-lineage of IOL CHIKV with E1-K211E, E2-V264A, and E1-226A.

A phylogenetic tree of nearly the whole genome shown in Figure 3 suggests that the adaptive E1-A226V substitution has independently occurred at least once in the ECSA-West African lineage, once in the ECSA-IOL sub-lineage of the Indian Ocean islands, and once in the ECSA-IOL sub-lineage of the Indian subcontinent/SEA. In contrast, other adaptive substitutions E1-K211E and E2-V264A have occurred only once in the new sub-lineage of the northern Indian subcontinent/SEA in India. Thus, it is important to monitor whether similar adaptive mutations occur in other geographical regions in other sub-lineages of the CHIKV ECSA genotype.

In conclusion, we found the new sub-lineages of ECSA IOL CHIKV carrying E1-K211E and E2-V264A, which have expanded from the northern Indian subcontinent to Thailand through Bangladesh. It is important to see whether this new sub-lineage of ECSA-IOL CHIKV could expand into other regions of the world.

## Figures and Tables

**Table 1:** CHIKVs in the present study.

Strain	Collection Date	Location	Country	Sample Type	Passage History	Accession No.
BGD17-0266	July 2017	Maghbazar, Dhaka	Bangladesh	serum	0	LC580236
BGD17-0290	July 2017	Uttara, Dhaka	Bangladesh	isolate	C6/36	LC580237
BGD17-0303	July 2017	Bashundhara, Dhaka	Bangladesh	serum	0	LC580238
BGD17-0306	July 2017	Maghbazar, Dhaka	Bangladesh	serum	0	LC580239
BGD17-0932	August 2017	Bashundhara, Dhaka	Bangladesh	serum	0	LC580240
BGD17-1024	September 2017	Bashundhara, Dhaka	Bangladesh	serum	0	LC580241
BGD17-1147	September 2017	Bashundhara, Dhaka	Bangladesh	serum	0	LC580242
BGD17-1151	September 2017	Bashundhara, Dhaka	Bangladesh	isolate	C6/36	LC580243
BGD17-1210	September 2017	Bashundhara, Dhaka	Bangladesh	isolate	C6/36	LC580244
BGD17-1232	September 2017	Bashundhara, Dhaka	Bangladesh	isolate	C6/36	LC580245
BGD17-1269	September 2017	Bashundhara, Dhaka	Bangladesh	isolate	C6/36	LC580246
BGD17-1270	September 2017	Bashundhara, Dhaka	Bangladesh	isolate	C6/36	LC580247
BGD17-1299	October 2017	Rampura, Dhaka	Bangladesh	isolate	C6/36	LC580248
BGD17-1319	October 2017	Chittagon	Bangladesh	isolate	C6/36	LC580249
BGD17-1332	October 2017	Mirpur, Dhaka	Bangladesh	serum	0	LC580250
BGD17-1413	October 2017	Mirpur, Dhaka	Bangladesh	serum	0	LC580251
BGD17-1457	October 2017	Gulshan, Dhaka	Bangladesh	isolate	C6/36	LC580252
BGD17-1538	November 2017	Uttara, Dhaka	Bangladesh	serum	0	LC580253
BGD17-1542	November 2017	Gulshan, Dhaka	Bangladesh	serum	0	LC580254
BGD17-1629	December 2017	Uttara, Dhaka	Bangladesh	serum	0	LC580255
THBKK19-04	October 2019	Bangkok	Thailand	serum	0	LC580256
THBKK19-06	October 2019	Bangkok	Thailand	isolate	C6/36	LC580257
THBKK19-08	October 2019	Bangkok	Thailand	serum	0	LC580258
THBKK19-09	October 2019	Bangkok	Thailand	isolate	C6/36	LC580259
THBKK19-11	October 2019	Bangkok	Thailand	isolate	C6/36	LC580260
THBKK19-12	October 2019	Bangkok	Thailand	isolate	C6/36	LC580261
THBKK19-13	October 2019	Bangkok	Thailand	serum	0	LC580262
THBKK19-14	October 2019	Bangkok	Thailand	serum	0	LC580263
THBKK19-15	October 2019	Bangkok	Thailand	isolate	C6/36	LC580264
THBKK19-16	October 2019	Bangkok	Thailand	isolate	C6/36	LC580265
THBKK19-17	October 2019	Bangkok	Thailand	isolate	C6/36	LC580266
THBKK19-19	October 2019	Bangkok	Thailand	serum	0	LC580267
THBKK19-20	October 2019	Bangkok	Thailand	serum	0	LC580268
THBKK19-21	October 2019	Bangkok	Thailand	serum	0	LC580269
CP10	2010	Ratchaburi	Thailand	isolate	C6/36 and Vero	LC580270

**Table 2:** tMRCA and evolution rates of ECSA-IOL CHIKV lineages.

Node	Lineage/Clade	Detected Areas/Countries	PP	tMRCA (95% HPD)	Substitution Rate × 10^−4^ (95% HPD)
Root	Root ECSA		1	1949.17 (1944.14–1957.42)	5.74 (4.96–6.60)
A	Enzootic ECSA	South Africa, Central African Republic, Senegal, and Tanzania	0.39	1950.48 (1948.58–1952.53)	5.70 (0.80–12.62)
B	African ECSA	Africa and Brazil	1	1970.39 (1967.68–1973.27)	5.60 (1.37–11.68)
B1	West African ECSA	Congo and Angola	1	2011.39 (2008.69–2014.26)	1.02 (0.69–1.36)
B2	South American ECSA	Brazil	1	2013.66 (2013.29–2014.18)	3.27 (2.59–3.92)
C	Indian Ocean Lineage (IOL)	Coastal Kenya, Indian Ocean Islands, Indian subcontinent, SEA countries, Italy, and France	1	2002.26 (2001.14–2003.59)	2.98 (2.31–3.67)
C1	Indian Ocean Islands Sub-lineage	Indian Ocean Islands, France	0.98	2003.92 (2003.43–2004.45)	2.88 (5.93–6.10)
C2	Indian subcontinent and SEA Sub-lineage	Indian subcontinent, SEA countries, and Italy	1	2004.16 (2003.52–2004.88)	4.72 (1.34–8.93)
C2.1	Indian subcontinent	India, Sri Lanka, and Bangladesh	0.81	2005.33 (2004.74–2006.15)	4.16 (0.61–9.57)
C2.2	Indian subcontinent/SEA	Indian Continent, SEA countries, and Italy	1	2004.97 (2004.46–2005.52)	6.91 (1.49–14.50)
C2.2a	SEA clade	Malaysia, Singapore, Thailand, Cambodia, and Laos	1	2006.82 (2006.42–2007.25)	4.91 (1.17–10.07)
C2.3	Northern Indian subcontinent/SEA	Northern Indian subcontinent, SEA countries, and Italy	1	2008.75 (2008.18–2009.37)	6.29 (1.57–13.03)
C2.3a	India clade	India, Pakistan, Bangladesh, Italy, Myanmar, and Thailand	1	2012.71 (2011.72–2013.74)	8.52 (2.52–16.22)
C2.3b	Bangladesh clade	Bangladesh, Italy, Myanmar, and Thailand	1	2015.61 (2015.25–2016.01)	6.79 (1.75–13.76)
C2.3c	Thailand clade	Thailand and Myanmar	1	2016.98 (2016.73–2017.25)	10.75 (3.07–20.41)

SEA= Southeast Asia.

**Table 3:** Nonsynonymous amino acid substitutions in CHIKV ECSA-IOL sub-lineages among CHIKV outbreak strains compared to the ancestor IOL.

Polyproteins	Position*	Protein	Position**	Ancestor IOL	Indian Ocean Islands (C1***)	Indian Sub-Continent (C2.1***)	Indian Sub-Continent/SEA (C2.2a***)	Northern Indian Subcontinent/SEA (C2.3***)					
				Kenya 2004	Reunion 2005	India 2006	Thailand 2010	India 2010	India 2016	Pakistan 2016	Bangladesh 2017	Italy 2017	Thailand 2019
Non-structural protein	665	nsP2	130	H	H	H	H	H	H/Y	Y	Y	Y	Y
	680	nsP2	145	E	E	E	E	E	E/D	D	D	D	D
	1030	nsP2	495	N	N	N	N	N	N	N	N	N	S
	1328	nsP2	793	V	V	V	V	V	V/I	V	V/A	V	A
	1391	nsP3	58	V	V	V	V	V	V	V	V/I	V	V
	1705	nsP3	372	D	D	D	D	D	D	D	E	E	E
	1918	nsP4	55	S	S	S	S	S	S/N	N	N	N	N
	1948	nsP4	85	R	R	R	R	R	R/G	G	G	G	G
Structural protein	73	Capsid	73	K	K	K	K	K	K	K	K	K	R
	530	E2	205	G	G	G	G	G	G	G	S	S	S
	546	E2	221	K	K	K	K	K	K	K	K/R	K	K
	589	E2	264	V	V	V	V	A	A	A	A	A	A
	1020	E1	211	K	K	K	K	E	E	E	E	E	E
	1035	E1	226	A	A/V	A/V	V	A	A	A	A	A	A
	1126	E1	317	I	I	I	I	I	I/V	V	V	V	V

* Codon numbering from the first codon in each open reading frame, ** codon numbering from the first codon in each viral protein, *** corresponds to the defined nodes in Figure 3B and Table 2.

**Table 4:** Codon sites and amino acid substitutions under positive selection pressure.

Dataset	FEL	FUBAR	MEME	
ECSA dataset	171: nsP1-R171Q 488: nsP1-Q488R 1550: nsP3-H217Y 1695: nsP3-A362V 2418: nsP4-V555I 1020: E1-E211K	171: nsP-R171Q 1550: nsP3-H217Y 1661: nsP3-Q328P 2418: nsP4-V555I 471: E2-Q146R 1020: E1-E211K	82: nsP1-C82S 171: nsP1-R171Q 178: nsP1-F178V 753: nsP2-T218I 992: nsP2-I457S 1426: nsP3-A93P 1550: nsP3-H217Y 1661: nsP3-Q328P 1768: nsP3-C435R 1849: nsP3-C516L 2325: nsP4-A462G 2330: nsP4-D467Y	147: C-A147R 156: C-R156A 467: E2-H142Y 471: E2-Q146R 632: E2-Q307R 795: 6K-A47G/V 955: E1-A146N 1020: E1-E211K 1100: E1-V291I 1191: E1-P382F
The present study dataset	1030: nsP2-N495S 1391: nsP3-V58I 1550: nsP3-H217Y 1695: nsP3-A362V 2228: nsP4-T365A 73: C-K73R 546: E2-K221R	1391: nsP3-V58I 73: C-K73R		

Amino acid positions are within either the nonstructural (ns) or structural polyprotein.

## Supplementary Materials

The following are available online at https://www.mdpi.com/1999-4915/12/11/1319/s1, Table S1. CHIKV sequences in the present study; Table S2. Model-fit comparison of the log marginal likelihood estimation; Figure S1. Maximum clade credibility (MCC) tree of ECSA-genotype CHIKV ORFs

## References

[1] Halstead S.B. (2015). Reappearance of chikungunya, formerly called dengue, in the Americas. Emerg. Infect. Dis..

[2] Volk S.M., Chen R., Tsetsarkin K.A., Adams A.P., Garcia T.I., Sall A.A., Nasar F., Schuh A.J., Holmes E.C., Higgs S. (2010). Genome-scale phylogenetic analyses of chikungunya virus reveal independent emergences of recent epidemics and various evolutionary rates. J. Virol..

[3] Weaver S.C., Chen R., Diallo M. (2020). Chikungunya Virus: Role of Vectors in Emergence from Enzootic Cycles. Annu. Rev. Entomol..

[4] Weaver S.C. (2014). Arrival of chikungunya virus in the new world: Prospects for spread and impact on public health. PLoS Negl. Trop. Dis..

[5] Schuffenecker I., Iteman I., Michault A., Murri S., Frangeul L., Vaney M.C., Lavenir R., Pardigon N., Reynes J.M., Pettinelli F. (2006). Genome microevolution of chikungunya viruses causing the Indian Ocean outbreak. PLoS Med..

[6] Tsetsarkin K.A., Vanlandingham D.L., McGee C.E., Higgs S. (2007). A single mutation in chikungunya virus affects vector specificity and epidemic potential. PLoS Pathog..

[7] Tan Y., Pickett B.E., Shrivastava S., Gresh L., Balmaseda A., Amedeo P., Hu L., Puri V., Fedorova N.B., Halpin R.A. (2018). Differing epidemiological dynamics of Chikungunya virus in the Americas during the 2014-2015 epidemic. PLoS Negl. Trop. Dis..

[8] Nunes M.R., Faria N.R., de Vasconcelos J.M., Golding N., Kraemer M.U., de Oliveira L.F., Azevedo Rdo S., da Silva D.E., da Silva E.V., da Silva S.P. (2015). Emergence and potential for spread of Chikungunya virus in Brazil. BMC Med..

[9] Jain J., Kaur N., Haller S.L., Kumar A., Rossi S.L., Narayanan V., Kumar D., Gaind R., Weaver S.C., Auguste A.J. (2020). Chikungunya Outbreaks in India: A Prospective Study Comparing Neutralization and Sequelae during Two Outbreaks in 2010 and 2016. Am. J. Trop. Med. Hyg..

[10] Shrinet J., Jain S., Sharma A., Singh S.S., Mathur K., Rana V., Bhatnagar R.K., Gupta B., Gaind R., Deb M. (2012). Genetic characterization of Chikungunya virus from New Delhi reveal emergence of a new molecular signature in Indian isolates. Virol. J..

[11] Rahman M., Yamagishi J., Rahim R., Hasan A., Sobhan A. (2019). East/Central/South African Genotype in a Chikungunya Outbreak, Dhaka, Bangladesh, 2017. Emerg. Infect. Dis..

[12] Aamir U.B., Badar N., Salman M., Ahmed M., Alam M.M. (2017). Outbreaks of chikungunya in Pakistan. Lancet Infect. Dis..

[13] Venturi G., Di Luca M., Fortuna C., Remoli M.E., Riccardo F., Severini F., Toma L., Del Manso M., Benedetti E., Caporali M.G. (2017). Detection of a chikungunya outbreak in Central Italy, August to September 2017. Eurosurveillance.

[14] Maljkovic Berry I., Eyase F., Pollett S., Konongoi S.L., Joyce M.G., Figueroa K., Ofula V., Koka H., Koskei E., Nyunja A. (2019). Global Outbreaks and Origins of a Chikungunya Virus Variant Carrying Mutations Which May Increase Fitness for Aedes aegypti: Revelations from the 2016 Mandera, Kenya Outbreak. Am. J. Trop. Med. Hyg..

[15] BOE, National Disease Surveillance (Report 506) Chikungunya: Bureau of Epidemiology, Ministry of Public Health, Thailand. http://www.boe.moph.go.th/boedb/surdata/disease.php?ds=84.

[16] Suzuki K., Huits R., Phadungsombat J., Tuekprakhon A., Nakayama E.E., van den Berg R., Barbe B., Cnops L., Rahim R., Hasan A. (2020). Promising application of monoclonal antibody against chikungunya virus E1-antigen across genotypes in immunochromatographic rapid diagnostic tests. Virol. J..

[17] Sasayama M., Benjathummarak S., Kawashita N., Rukmanee P., Sangmukdanun S., Masrinoul P., Pitaksajjakul P., Puiprom O., Wuthisen P., Kurosu T. (2014). Chikungunya virus was isolated in Thailand, 2010. Virus Genes.

[18] Phadungsombat J., Tuekprakhon A., Cnops L., Michiels J., van den Berg R., Nakayama E.E., Shioda T., Arien K.K., Huits R. (2020). Two distinct lineages of chikungunya virus cocirculated in Aruba during the 2014-2015 epidemic. Infect. Genet. Evol..

[19] Pyke A.T., McMahon J., Burtonclay P., Nair N., De Jong A. (2020). Genome Sequences of Chikungunya Virus Strains from Bangladesh and Thailand. Microbiol. Resour. Announc..

[20] Pickett B.E., Sadat E.L., Zhang Y., Noronha J.M., Squires R.B., Hunt V., Liu M., Kumar S., Zaremba S., Gu Z. (2012). ViPR: An open bioinformatics database and analysis resource for virology research. Nucleic. Acids Res..

[21] Arankalle V.A., Shrivastava S., Cherian S., Gunjikar R.S., Walimbe A.M., Jadhav S.M., Sudeep A.B., Mishra A.C. (2007). Genetic divergence of Chikungunya viruses in India (1963-2006) with special reference to the 2005–2006 explosive epidemic. J. Gen. Virol..

[22] Cherian S.S., Walimbe A.M., Jadhav S.M., Gandhe S.S., Hundekar S.L., Mishra A.C., Arankalle V.A. (2009). Evolutionary rates and timescale comparison of Chikungunya viruses inferred from the whole genome/E1 gene with special reference to the 2005-07 outbreak in the Indian subcontinent. Infect. Genet. Evol..

[23] Larsson A. (2014). AliView: A fast and lightweight alignment viewer and editor for large datasets. Bioinformatics.

[24] Trifinopoulos J., Nguyen L.T., von Haeseler A., Minh B.Q. (2016). W-IQ-TREE: A fast online phylogenetic tool for maximum likelihood analysis. Nucleic. Acids Res..

[25] Hoang D.T., Chernomor O., von Haeseler A., Minh B.Q., Vinh L.S. (2018). UFBoot2: Improving the Ultrafast Bootstrap Approximation. Mol. Biol. Evol..

[26] Kalyaanamoorthy S., Minh B.Q., Wong T.K.F., von Haeseler A., Jermiin L.S. (2017). ModelFinder: Fast model selection for accurate phylogenetic estimates. Nat. Methods.

[27] Rambaut A., Lam T.T., Max Carvalho L., Pybus O.G. (2016). Exploring the temporal structure of heterochronous sequences using TempEst (formerly Path-O-Gen). Virus. Evol..

[28] Suchard M.A., Lemey P., Baele G., Ayres D.L., Drummond A.J., Rambaut A. (2018). Bayesian phylogenetic and phylodynamic data integration using BEAST 1.10. Virus Evol..

[29] Baele G., Lemey P., Suchard M.A. (2016). Genealogical Working Distributions for Bayesian Model Testing with Phylogenetic Uncertainty. Syst. Biol..

[30] Shapiro B., Rambaut A., Drummond A.J. (2006). Choosing appropriate substitution models for the phylogenetic analysis of protein-coding sequences. Mol. Biol. Evol..

[31] Gill M.S., Lemey P., Faria N.R., Rambaut A., Shapiro B., Suchard M.A. (2013). Improving Bayesian population dynamics inference: A coalescent-based model for multiple loci. Mol. Biol. Evol..

[32] Weaver S., Shank S.D., Spielman S.J., Li M., Muse S.V., Kosakovsky Pond S.L. (2018). Datamonkey 2.0: A Modern Web Application for Characterizing Selective and Other Evolutionary Processes. Mol. Biol. Evol..

[33] Murrell B., Wertheim J.O., Moola S., Weighill T., Scheffler K., Kosakovsky Pond S.L. (2012). Detecting individual sites subject to episodic diversifying selection. PLoS Genet.

[34] Murrell B., Moola S., Mabona A., Weighill T., Sheward D., Kosakovsky Pond S.L., Scheffler K. (2013). FUBAR: A fast, unconstrained bayesian approximation for inferring selection. Mol. Biol. Evol..

[35] Kosakovsky Pond S.L., Frost S.D. (2005). Not so different after all: A comparison of methods for detecting amino acid sites under selection. Mol. Biol. Evol..

[36] Kariuki Njenga M., Nderitu L., Ledermann J.P., Ndirangu A., Logue C.H., Kelly C.H.L., Sang R., Sergon K., Breiman R., Powers A.M. (2008). Tracking epidemic Chikungunya virus into the Indian Ocean from East Africa. J. Gen. Virol..

[37] Kabir I., Dhimal M., Muller R., Banik S., Haque U. (2017). The 2017 Dhaka chikungunya outbreak. Lancet Infect. Dis..

[38] Diaz-Menendez M., Esteban E.T., Ujiie M., Calleri G., Rothe C., Malvy D., Nicastri E., Bissinger A.L., Grandadam M., Alpern J.D. (2020). Travel-associated chikungunya acquired in Myanmar in 2019. Eurosurveillance.

[39] Venturi G., Aberle S.W., Avsic-Zupanc T., Barzon L., Batejat C., Burdino E., Carletti F., Charrel R., Christova I., Connell J. (2020). Specialist laboratory networks as preparedness and response tool the Emerging Viral Diseases-Expert Laboratory Network and the Chikungunya outbreak, Thailand, 2019. Eurosurveillance.

[40] Zakotnik S., Korva M., Knap N., Robnik B., Gorisek Miksic N., Avsic Zupanc T. (2019). Complete Coding Sequence of a Chikungunya Virus Strain Imported into Slovenia from Thailand in Late 2018. Microbiol. Resour. Announc..

[41] Chen R., Puri V., Fedorova N., Lin D., Hari K.L., Jain R., Rodas J.D., Das S.R., Shabman R.S., Weaver S.C. (2016). Comprehensive Genome Scale Phylogenetic Study Provides New Insights on the Global Expansion of Chikungunya Virus. J. Virol..

[42] Vazeille M., Moutailler S., Coudrier D., Rousseaux C., Khun H., Huerre M., Thiria J., Dehecq J.S., Fontenille D., Schuffenecker I. (2007). Two Chikungunya isolates from the outbreak of La Reunion (Indian Ocean) exhibit different patterns of infection in the mosquito, Aedes albopictus. PLoS ONE.

[43] Naresh Kumar C.V., Sivaprasad Y., Sai Gopal D.V. (2016). Genetic diversity of 2006-2009 Chikungunya virus outbreaks in Andhra Pradesh, India, reveals complete absence of E1:A226V mutation. Acta Virol..

[44] Sumathy K., Ella K.M. (2012). Genetic diversity of Chikungunya virus, India 2006-2010: Evolutionary dynamics and serotype analyses. J. Med. Virol..

[45] Abraham R., Manakkadan A., Mudaliar P., Joseph I., Sivakumar K.C., Nair R.R., Sreekumar E. (2016). Correlation of phylogenetic clade diversification and in vitro infectivity differences among Cosmopolitan genotype strains of Chikungunya virus. Infect. Genet Evol..

[46] Santhosh S.R., Dash P.K., Parida M.M., Khan M., Tiwari M., Lakshmana Rao P.V. (2008). Comparative full genome analysis revealed E1: A226V shift in 2007 Indian Chikungunya virus isolates. Virus Res..

[47] Taraphdar D., Chatterjee S. (2015). Molecular characterization of chikungunya virus circulating in urban and rural areas of West Bengal, India after its re-emergence in 2006. Trans. R Soc. Trop Med. Hyg..

[48] Rianthavorn P., Prianantathavorn K., Wuttirattanakowit N., Theamboonlers A., Poovorawan Y. (2010). An outbreak of chikungunya in southern Thailand from 2008 to 2009 caused by African strains with A226V mutation. Int. J. Infect. Dis..

[49] Hapuarachchi H.C., Bandara K.B., Sumanadasa S.D., Hapugoda M.D., Lai Y.L., Lee K.S., Tan L.K., Lin R.T., Ng L.F., Bucht G. (2010). Re-emergence of Chikungunya virus in South-east Asia: Virological evidence from Sri Lanka and Singapore. J. Gen. Virol..

[50] Newase P., More A., Patil J., Patil P., Jadhav S., Alagarasu K., Shah P., Parashar D., Cherian S.S. (2020). Chikungunya phylogeography reveals persistent global transmissions of the Indian Ocean Lineage from India in association with mutational fitness. Infect. Genet. Evol..

[51] Fahmy N.T., Klena J.D., Mohamed A.S., Zayed A., Villinski J.T. (2015). Complete Genome Sequence of Chikungunya Virus Isolated from an Aedes aegypti Mosquito during an Outbreak in Yemen, 2011. Genome Announc..

[52] Nyari N., Maan H.S., Sharma S., Pandey S.N., Dhole T.N. (2016). Identification and genetic characterization of chikungunya virus from Aedes mosquito vector collected in the Lucknow district, North India. Acta Trop..

[53] Agarwal A., Sharma A.K., Sukumaran D., Parida M., Dash P.K. (2016). Two novel epistatic mutations (E1:K211E and E2:V264A) in structural proteins of Chikungunya virus enhance fitness in Aedes aegypti. Virology.

[54] Haque F., Rahman M., Banu N.N., Sharif A.R., Jubayer S., Shamsuzzaman A., Alamgir A., Erasmus J.H., Guzman H., Forrester N. (2019). An epidemic of chikungunya in northwestern Bangladesh in 2011. PLoS ONE.

[55] Khatun S., Chakraborty A., Rahman M., Nasreen Banu N., Rahman M.M., Hasan S.M., Luby S.P., Gurley E.S. (2015). An Outbreak of Chikungunya in Rural Bangladesh, 2011. PLoS Negl. Trop. Dis..

[56] Anwar S., Taslem Mourosi J., Khan M.F., Ullah M.O., Vanakker O.M., Hosen M.J. (2020). Chikungunya outbreak in Bangladesh (2017): Clinical and hematological findings. PLoS Negl. Trop. Dis..

[57] Hossain M.S., Hasan M.M., Islam M.S., Islam S., Mozaffor M., Khan M.A.S., Ahmed N., Akhtar W., Chowdhury S., Arafat S.M.Y. (2018). Chikungunya outbreak (2017) in Bangladesh: Clinical profile, economic impact and quality of life during the acute phase of the disease. PLoS Negl. Trop. Dis..

[58] Paul K.K., Dhar-Chowdhury P., Haque C.E., Al-Amin H.M., Goswami D.R., Kafi M.A.H., Drebot M.A., Lindsay L.R., Ahsan G.U., Brooks W.A. (2018). Risk factors for the presence of dengue vector mosquitoes, and determinants of their prevalence and larval site selection in Dhaka, Bangladesh. PLoS ONE.

[59] Saha S., Ramesh A., Kalantar K., Malaker R., Hasanuzzaman M., Khan L.M., Mayday M.Y., Sajib M.S.I., Li L.M., Langelier C. (2019). Unbiased Metagenomic Sequencing for Pediatric Meningitis in Bangladesh Reveals Neuroinvasive Chikungunya Virus Outbreak and Other Unrealized Pathogens. mBio.

[60] Pan J., Fang C., Yan J., Yan H., Zhan B., Sun Y., Liu Y., Mao H., Cao G., Lv L. (2019). Chikungunya Fever Outbreak, Zhejiang Province, China, 2017. Emerg. Infect. Dis..

[61] Rezza G., Nicoletti L., Angelini R., Romi R., Finarelli A.C., Panning M., Cordioli P., Fortuna C., Boros S., Magurano F. (2007). Infection with chikungunya virus in Italy: An outbreak in a temperate region. Lancet.

[62] Fortuna C., Toma L., Remoli M.E., Amendola A., Severini F., Boccolini D., Romi R., Venturi G., Rezza G., Di Luca M. (2018). Vector competence of Aedes albopictus for the Indian Ocean lineage (IOL) chikungunya viruses of the 2007 and 2017 outbreaks in Italy: A comparison between strains with and without the E1:A226V mutation. Eurosurveillance.

[63] Intayot P., Phumee A., Boonserm R., Sor-Suwan S., Buathong R., Wacharapluesadee S., Brownell N., Poovorawan Y., Siriyasatien P. (2019). Genetic Characterization of Chikungunya Virus in Field-Caught Aedes aegypti Mosquitoes Collected during the Recent Outbreaks in 2019, Thailand. Pathogens.

[64] Thavara U., Tawatsin A., Pengsakul T., Bhakdeenuan P., Chanama S., Anantapreecha S., Molito C., Chompoosri J., Thammapalo S., Sawanpanyalert P. (2009). Outbreak of chikungunya fever in Thailand and virus detection in field population of vector mosquitoes, Aedes aegypti (L.) and Aedes albopictus Skuse (Diptera: Culicidae). Southeast Asian J. Trop. Med. Public Health.

