# Supplementary figures and images for: A Novel Sub-Lineage of Chikungunya Virus East/Central/South African Genotype Indian Ocean Lineage Caused Sequential Outbreaks in Bangladesh and Thailand
Source: Viruses. 2020 Nov 17;12(11):1319. doi: 10.3390/v12111319 (PMC7698486; doi:10.3390/v12111319)

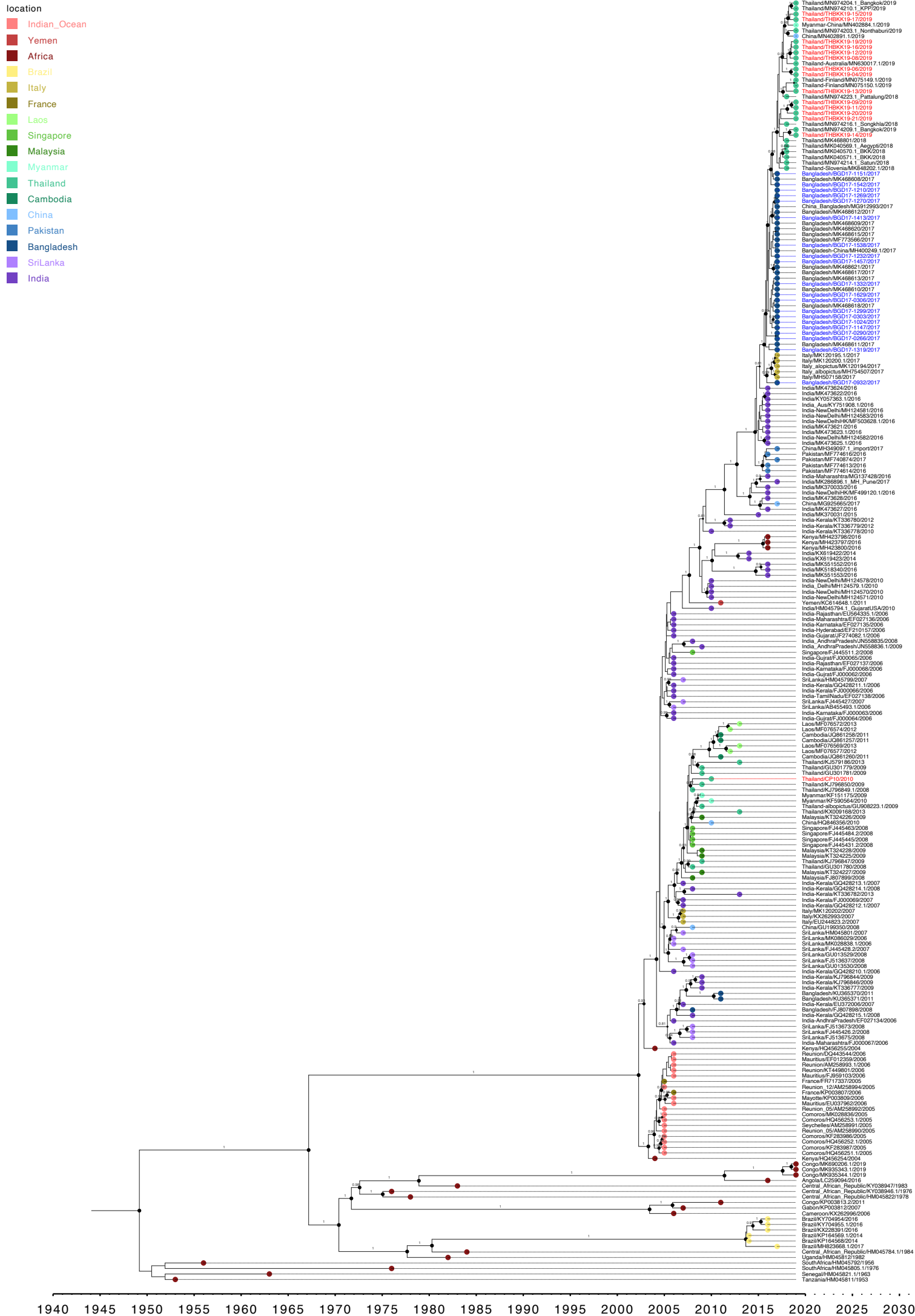

Supplement: Supplementary file 1 [file viruses-12-01319-s001.zip › Supplementary files/Fig S1_full tree.pdf]
